# High-resolution plasma metabolomics analysis to detect *Mycobacterium tuberculosis*-associated metabolites that distinguish active pulmonary tuberculosis in humans

**DOI:** 10.1371/journal.pone.0205398

**Published:** 2018-10-11

**Authors:** Jeffrey M. Collins, Douglas I. Walker, Dean P. Jones, Nestani Tukvadze, Ken H. Liu, ViLinh T. Tran, Karan Uppal, Jennifer K. Frediani, Kirk A. Easley, Neeta Shenvi, Manoj Khadka, Eric A. Ortlund, Russell R. Kempker, Henry M. Blumberg, Thomas R. Ziegler

**Affiliations:** 1 Division of Infectious Diseases, Department of Medicine, Emory University School of Medicine, Atlanta, Georgia, United States of America; 2 Department of Environmental Health, Rollins School of Public Health, Emory University, Atlanta, Georgia, United States of America; 3 Clinical Biomarkers Laboratory, Division of Pulmonary, Allergy, and Critical Care Medicine, Department of Medicine, Emory University School of Medicine, Atlanta, Georgia, United States of America; 4 National Center for Tuberculosis and Lung Disease, Tbilisi, Georgia; 5 Nell Hodgson Woodruff School of Nursing, Emory University, Atlanta, Georgia, United States of America; 6 Department of Biostatistics and Bioinformatics, Rollins School of Public Health, Emory University, Atlanta, Georgia, United States of America; 7 Emory Integrated Lipidomics Core, Department of Biochemistry, Emory University School of Medicine, Atlanta, Georgia, United States of America; 8 Division of Endocrinology, Metabolism and Lipids and Center for Clinical and Molecular Nutrition, Department of Medicine, Emory University School of Medicine, Atlanta, Georgia, United States of America; 9 Section of Endocrinology, Atlanta Veterans Affairs Medical Center, Atlanta, Georgia, United States of America; Rutgers Biomedical and Health Sciences, UNITED STATES

## Abstract

**Introduction:**

Pulmonary tuberculosis (TB) is a major worldwide health problem that lacks robust blood-based biomarkers for detection of active disease. High-resolution metabolomics (HRM) is an innovative method to discover low-abundance metabolites as putative blood biomarkers to detect TB disease, including those known to be produced by the causative organism, *Mycobacterium tuberculosis (Mtb)*.

**Methods:**

We used HRM profiling to measure the plasma metabolome for 17 adults with active pulmonary TB disease and 16 of their household contacts without active TB. We used a suspect screening approach to identify metabolites previously described in cell culture studies of *Mtb* based on retention time and accurate mass matches.

**Results:**

The association of relative metabolite abundance in active TB disease subjects compared to their household contacts predicted three *Mtb*-associated metabolites that were significantly increased in the active TB patients based on accurate mass matches: phosphatidylglycerol (PG) (16:0_18:1), lysophosphatidylinositol (Lyso-PI) (18:0) and acylphosphatidylinositol mannoside (Ac1PIM1) (56:1) (p<0.001 for all). These three metabolites provided excellent classification accuracy for active TB disease (AUC = 0.97). Ion dissociation spectra (tandem MS/MS) supported the identification of PG (16:0_18:1) and Lyso-PI (18:0) in the plasma of patients with active TB disease, though the identity of Ac1PIM1 could not be definitively confirmed.

**Conclusions:**

Presence of the *Mtb*-associated lipid metabolites PG (16:0_18:1) and Lyso-PI (18:0) in plasma accurately identified patients with active TB disease. Consistency of *in vitro* and *in vivo* data suggests suitability for exploring these in future studies for possible development as TB disease biomarkers.

## Introduction

Tuberculosis (TB) is a major global health problem and now the leading cause of death due to an infectious disease [1]. Rapid and accurate detection of active TB disease remains a major challenge in global control efforts, with less than two thirds of estimated TB cases diagnosed in 2016 [1]. The poor performance characteristics of available diagnostic tests for pulmonary TB, especially smear microscopy, which is frequently used in low- and middle-income countries, is a major contributor to suboptimal case detection and diagnostic delays [2]. Acid-fast bacilli (AFB) culture is the gold standard for diagnosis, but can take 2–6 weeks for definitive results and the infrastructure required for culture is often unavailable in resource-limited settings [3]. Furthermore, all tests require analysis of sputum specimens, which many adult patients and most children cannot adequately produce. There is an urgent need for point-of-care diagnostic tests for active TB which can be performed on peripheral blood or urine samples [3]. The development of such tests will initially require identification of new putative biomarkers in the peripheral blood associated with active TB disease [4, 5].

Recently developed high-resolution metabolomics (HRM) methods utilize liquid chromatography and ultra-high-resolution mass spectrometry (LC-MS), coupled with advanced methods in data extraction and bioinformatics, to detect tens of thousands of metabolites in plasma and other biosamples [6–8]. The high sensitivity of HRM allows for simultaneous detection and semi-quantitative measurement of multiple low-abundance metabolites, including those potentially derived from infecting microorganisms such as *Mycobacterium tuberculosis* (*Mtb)* [7, 8]. Yet the high number of chemical features detected creates challenges for comprehensive metabolite annotation. It is now apparent that searchable metabolite databases contain only a small fraction of various metabolites derived from humans, microorganisms and environmental chemicals; thus, many features detected by HRM are not identified and remain unknown chemicals [7, 9, 10]. Recent studies, including our own, have focused on untargeted (discovery) evaluation of changes in systemic endogenously-derived metabolites associated with active TB disease compared to controls without active TB [7, 11, 12]. However, the current sensitivity of HRM methods now enables the characterization of low-abundance metabolites in the human host, some of which may be pathogen-derived with the potential to provide new insights into host-pathogen interactions and pathophysiology.

In our preliminary studies using plasma HRM, we used available open-access human metabolite databases for metabolite annotation [13–15], with nearly half of the 61 metabolites differentiating TB cases from household contacts lacking matches to known metabolites based on accurate mass/charge (*m/z*) ratio and predicted adduct forms [7]. Because existing databases contain few metabolites derived from *Mtb*, we hypothesized that some of these “unknown” metabolites may be derived from *Mtb* in the infected host. Recently, using a combined LC-MS and computational approach, databases of metabolites derived from *Mtb* have been developed [16, 17]. We used these databases in a suspect screening approach to re-analyze the plasma HRM data from our previous investigation [7] for evidence of *Mtb*-associated metabolites in plasma that differentiate patients with newly diagnosed active pulmonary TB from asymptomatic household contacts without active TB.

## Methods

### Study design

We performed a cross-sectional analysis of plasma HRM profiles for 17 patients with active TB disease selected from a randomized, controlled trial of high-dose cholecalciferol treatment of patients with pulmonary TB conducted in the country of Georgia (clinicaltrials.gov identifier NCT00918086) [18]. Inclusion criteria for patients included age ≥ 18 years, newly diagnosed, symptomatic active TB disease, suggested by a positive AFB sputum smear and confirmed by semi-quantitative sputum culture for *Mtb* (performed at the Georgian National TB Reference Laboratory (NRL) [18]. Patients with other localizing symptoms were evaluated for the presence of extrapulmonary TB. Plasma for HRM was obtained from eligible subjects within 7 days of initiating anti-TB drug therapy with first-line anti-TB drugs (isoniazid, rifampicin, pyrazinamide and ethambutol) [18]. Drug susceptibility testing (DST) was performed on all persons with pulmonary TB using the absolute concentration method. Patients infected with *Mtb* isolates resistant to isoniazid and rifampin were considered to have multidrug resistant (MDR)-TB.

The control group consisted of 16 household contacts enrolled from persons accompanying patients to the TB treatment facility at the time of study enrollment (one household contact was excluded from analysis for technical reasons). To be eligible for enrollment, household contacts were required to have both a negative screen for clinical symptoms suggestive of active TB or other acute illness and a negative AFB sputum smear and culture. Household contacts were not evaluated with a chest radiograph and were not tested for latent TB infection (LTBI).

### Plasma sample collection

Peripheral blood samples were obtained by venipuncture from all subjects with TB disease and the household contacts upon entry into the study [18]. Blood was collected in ethylenediaminetetraacetic acid (EDTA)-containing tubes and centrifuged; isolated plasma was immediately frozen and stored at -80ºC. Samples were subsequently shipped on dry ice from the NRL in Tbilisi, Georgia to Emory University, Atlanta, GA, USA. Samples remained frozen during transit and were kept at -80ºC prior to metabolomics analysis.

### Plasma metabolomics analysis

Thawed plasma (65 μL) was treated with 130 μl acetonitrile (2:1, v/v) containing an internal isotopic standard mixture (3.5 μL/sample), as previously described [7,19]. The internal standard mix for quality control consisted of 14 stable isotopic chemicals covering a broad range of small molecules [19]. Samples were mixed and placed on ice for 30 min prior to centrifugation to remove protein. The resulting supernatant was transferred to low-volume autosampler vials maintained at 4ºC and analyzed in triplicate using a LTQ-Velos Orbitrap mass spectrometer (Thermo Scientific, San Jose, CA, USA) and C_18_ chromatography (Higgins Analytical, Targa, Mountain View, CA, USA, 2.1 x 10 cm) with formic acid/acetonitrile gradient [7]. The high-resolution mass spectrometer was operated in positive electrospray ionization mode over scan range of 85 to 2000 *m/z* and stored as .Raw files [6, 7]. Data were extracted and aligned using apLCMS [20] and xMSanalyzer [21] with each feature defined by specific *m/z* value, retention time and integrated ion intensity. Non-zero ion intensity values from technical replicates were averaged to create a single mean intensity value for each participant.

### Metabolite identification

A suspect screening approach was used to identify potential *Mtb* lipid metabolites present in the plasma samples from subjects with active TB disease and household contacts without TB. A previously published *Mtb* lipid library, “*Mtb* LipidDB” was used to annotate *Mtb* lipid candidates based on accurate mass matches to specific *m/z* in the *Mtb* library [16]. The database search included three metabolite (M) adducts for each lipid species *(m/z)*: plus hydrogen (H), plus sodium (Na), and minus water (H_2_O)/plus hydrogen, respectively (M+H, M+Na, and M-H_2_O+H), with a mass accuracy threshold of 10 parts-per-million (ppm).

For those *Mtb* lipid metabolite suspects found to be significantly increased in patients with active TB disease relative to household contacts, we attempted to verify their chemical identities using ion dissociation methods (MS/MS). To maximize the ability to detect interpretable fragmentation spectra for lipid species of interest, additional plasma samples were extracted by adding 400 μl methanol followed by addition of a methyl *tert*-butyl ether (MTBE):methanol (1:1 v/v) mixture, and finally with 500 μl of 100% MTBE. All extract fractions were pooled and dried under a gentle stream of nitrogen. The dried extracts were resolvated in 500 μl of a chloroform:methanol (1:1 v/v) mixture followed by direct infusion analysis using a AB SCIEX QTRAP 5500 triple quadrupole mass spectrometer in product ion scan mode. MS/MS fragmentation of [M-H] adducts was performed in negative ionization mode. The MS/MS spectra were then investigated to find the lipid-specific fragments to confirm the chemical identity of the metabolites of interest.

### Data analysis

All analyses were performed in R version 3.3.0. Comparisons of descriptive statistics and clinical characteristics between subjects with pulmonary TB and their household contacts were performed using a Wilcoxon rank sum test for continuous variables and a Fisher exact test for categorical variables. The intensity profiles for *Mtb* lipid suspects were log_2_ transformed and tested for association with disease status using linear model for microarray data (LIMMA) analysis [22]. A Benjamini-Hochberg false discovery rate (FDR) of 20% was then applied to adjust for multiple comparisons and to identify those metabolites of greatest interest [23]. Logistic regression analysis was used to construct receiver operating characteristic (ROC) curves to determine the classification accuracy of the metabolites with the greatest -log P value.

### IRB approval

This study was approved by the Institutional Review Board (IRB) of Emory University (Atlanta, GA, USA) and the Georgian National Center for Tuberculosis and Lung Disease (NCTLD) Ethics Committee (Tbilisi, Georgia). All subjects provided written informed consent.

## Results

### Participants

Sex did not differ significantly in the 17 active TB cases compared to the 16 household contacts (Table 1). Those with active TB were more likely to smoke compared to the household contacts (76% vs 38%, *p* = 0.02) and had a lower median age (27 vs 35 years, *p* = 0.05). None of the patients with pulmonary TB had evidence of extrapulmonary disease. Three (18%) of the TB patients had MDR-TB and two (12%) had evidence of cavitation on chest x-ray. None of the subjects had HIV co-infection.

**Table 1:** Characteristics of patients diagnosed with pulmonary tuberculosis and their asymptomatic household contacts.

	Active TB Disease (n = 17)	Household Contacts (n = 16)	P-value^c^
**Age [years; median (IQR)]**^a^	27 (24–42)	35 (29–48)	0.05
**Male sex, n (%)**	10 (59%)	6 (38%)	0.22
**Tobacco use**	13 (76%)	6 (38%)	0.02
**High grade AFB sputum smear**^a^	1 (6%)	N/A	N/A
**>100 Colonies on semi-quantitative culture**	7 (41%)	N/A	N/A
**Cavitation on chest radiograph**	2 (12%)	N/A	N/A
**Multidrug-resistant TB**^b^	3 (18%)	N/A	N/A

IQR, interquartile range; AFB, acid-fast bacilli; TB, tuberculosis.

a. Sputum smear and culture obtained at study enrollment; high grade smear defined as > 1 AFB per high power field

b. Multidrug resistance was defined as resistance to both isoniazid and rifampin by drug susceptibility testing

c. A Wilcoxon rank sum test was used for comparisons of continuous data and a two-tailed Fisher exact test was used for categorical data

### High-resolution metabolomics profiling results

Following data extraction and alignment with apLCMS and xMSanalyzer, 33,262 mass spectral features, defined by accurate mass *m/z*, chromatographic retention time and intensity were detected. To maximize detection of low-abundance metabolites, three technical replicates were performed, and we removed features with a coefficient of variation (CV) ≥ 100% between technical replicates, resulting in 32,975 remaining features. The *m/z* values were then tested for accurate mass matches to the 1,696 unique lipid species present in *Mtb* LipidDB with adduct masses within the instrument scan range (85–2000 da) [16]. Using the criteria above, 2,470 matches to *Mtb* lipid suspects were detected by HRM (including M+H, M+Na, and M-H2O+H adducts of each specific *m/z* species). To target reliably detectable, non-polar ions consistent with *Mtb* lipids, we conservatively removed features exhibiting retention time ≤ 3 minutes and those with ≥ 50% missing values in both individuals with active TB disease and the household contacts, resulting in 867 remaining *Mtb* metabolite suspects.

**Fig 1:**
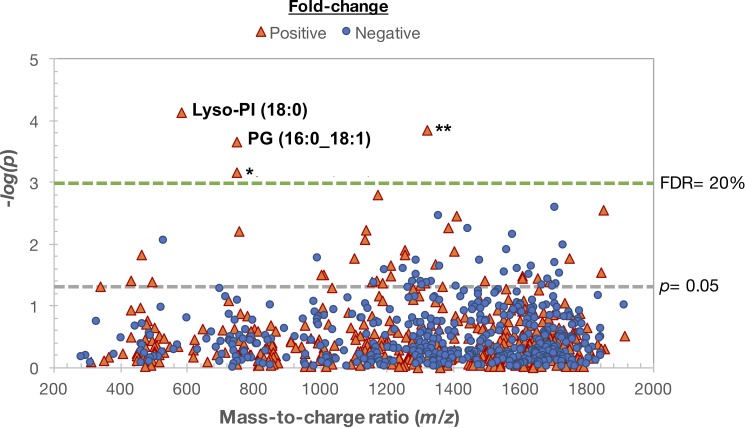
*Mycobacterium tuberculosis* (*Mtb)* lipid suspects in plasma of adults with active pulmonary TB compared to their household contacts. The Manhattan plot depicts the -log *p* statistical analysis of 867 features identified using plasma high-resolution metabolomics (HRM) as *Mtb*-associated metabolite suspects based on accurate mass/charge (*m/z)* matches. Analysis was done in a cross-sectional comparison of plasma from 17 adults with sputum culture-proven TB disease (within 7 days of diagnosis and initiation of anti-TB drugs) and 16 of their asymptomatic adult household contacts, who were sputum smear and culture negative for *Mtb*. Sixty-nine metabolites were significant at raw *p* ≤ 0.05 (points above gray line) and four were significant using a more stringent false discovery rate (FDR) threshold of 20% (red triangles above green line). The negative log_10_ statistical p-value of metabolites between the two groups are shown on the y-axis as a function of metabolite *m/z* (x-axis). *Chemical verification experiments revealed the metabolite with *m/z* 749.3714 was likely a source fragment of 25-desacetyl rifampin. **m/z 1321.9177 was predicted as acylphosphatidylinositol mannoside (Ac1PIM1) (56:1) based on accurate mass and retention time measurements, but could not be confirmed using tandem MS/MS; PG, phosphatidylglycerol; Lyso-PI, lysophostphatidylinositol.

**Fig 2:**
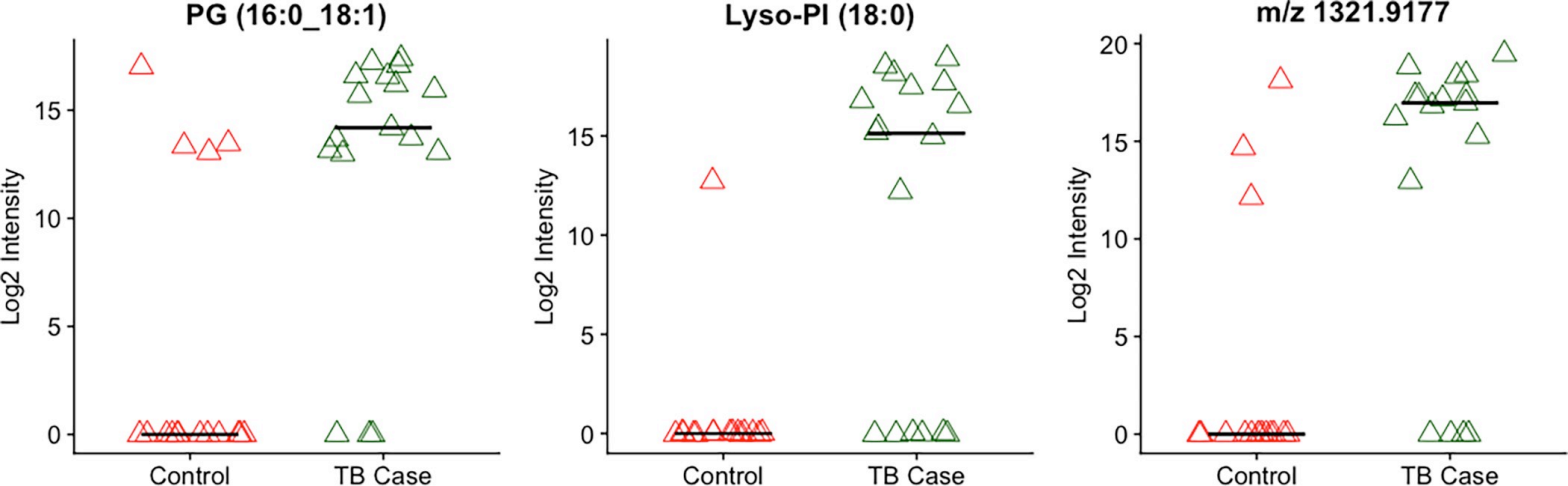
Three most significant *Mtb* lipid matches in plasma. Intensity values of the *Mtb*-associated metabolites phosphatidylglycerol (PG) (16:0_18:1) and lysophosphatidylinositol (Lyso-PI) (18:0), as well as m/z 1321.9177 [each *p* < 0.001; active TB cases (green triangles) vs household contacts (red triangles)]. *m/z* 1321.9177 was predicted as acylphosphatidylinositol mannoside (Ac1PIM1) (56:1) based on accurate mass and retention time measurements, but could not be confirmed using tandem MS/MS. Line depicts median values for each subject cohort.

Using LIMMA to test the association of log_2_ transformed intensity values of *Mtb* metabolite matches with active TB disease status, we obtained 69 suspects with *p* ≤ 0.05 (Fig 1). Application of an FDR correction of ≤ 20% [23] predicted four *Mtb*-associated metabolites significantly increased in patients with active TB and undetectable in nearly all household contacts (*p*<0.001 for all). Chemical verification experiments for these metabolites using MS/MS revealed that one was likely a source fragment of the rifampin metabolite 25-deacetylrifampin. The remaining three significant metabolites were predicted to be phosphatidylglycerol (PG) (16:0_18:1), lysophosphatidylinositol (Lyso-PI) (18:0) and acylphosphatidylinositol mannoside (Ac1PIM1) (56:1) based on accurate *m/z* and retention time measurements (Fig 2). At least one of these three metabolites was present in plasma of all persons with active TB disease and two or more were detectable in 14 of 17 patients with TB (82%). Among the 16 household contacts, one of the three metabolites was detectable in eight individuals (50%), and only one household contact (6%) had two of these metabolites detectable. Ion fragmentation spectra generated using tandem MS/MS supported the identification of PG (16:0_18:1) and Lyso-PI (18:0) in the plasma of active TB patients (S1 and S2 Figs), while a confirmatory fragmentation spectrum for Ac1PIM1 could not be generated.

### TB disease classification accuracy

**Fig 3:**
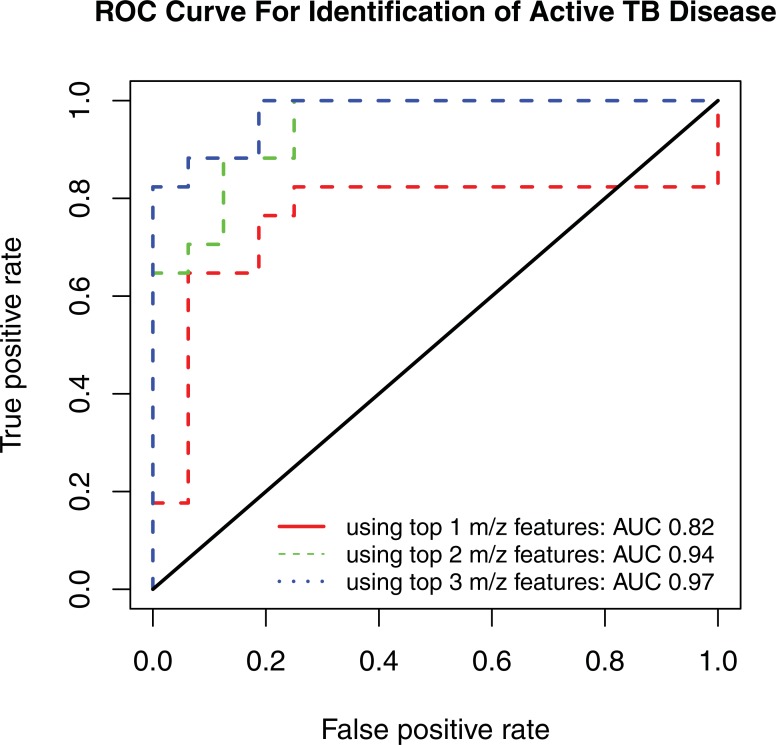
Receiver operating characteristic curve. *M*. *tuberculosis* lipid suspects phosphatidylglycerol (PG) (16:0_18:1), lysophosphatidylinositol (Lyso-PI) (18:0), and *m/z* 1321.9177 provided excellent classification accuracy for active TB disease [area under the curve (AUC) = 0.97], compared with PG (16:0_18:1) and Lyso-PI (18:0) (AUC = 0.94) or PG (16:0_18:1) alone (AUC = 0.82).

Using a logistic regression model, the log_2_ intensity value of the three most significant metabolites [PG (16:0_18:1), Lyso-PI (18:0) and *m/z* 1321.9177] provided excellent classification accuracy of active TB cases. The ROC curve demonstrated an area under the curve (AUC) of 0.97 (95% CI 0.93–1) (Table 2). The AUC was reduced to 0.94 when using only PG (16:0_18:1) and Lyso-PI (18:0) and 0.82 when PG (16:0_18:1) alone (Fig 3).

**Table 2:** Area under the receiver operator characteristic curve for varying combinations of *Mycobacterium tuberculosis* lipid suspects.

Metabolites	Area Under the Curve	95% Confidence Interval
**PG (16:0_18:1)**	0.82	0.68–0.97
**PG (16:0_18:1), Lyso-PI (18:0)**	0.94	0.88–1
PG (16:0_18:1), Lyso-PI (18:0), *m/z* 1321.9177*	0.97	0.93–1

* *m/z* 1321.9177 was predicted as acylphosphatidylinositol mannoside (Ac1PIM1) (56:1) based on accurate mass and retention time measurements, but could not be confirmed using tandem MS/MS; PG, phosphatidylglycerol; Lyso-PI, lysophosphatidylinositol

## Discussion

Using a suspect screening analysis of *Mtb*-associated metabolite features, we found that metabolites matching the mass and expected retention times of PG (16:0_18:1), Lyso-PI (18:0), and Ac1PIM1 (56:1) were the most significantly increased in the plasma of patients with active pulmonary TB and were largely absent from their asymptomatic household contacts without active TB. The identities of the PG (16:0_18:1) and Lyso-PI (18:0) were supported by tandem MS/MS analysis. Alone or together, these three metabolites demonstrated excellent classification accuracy for active TB cases (AUC 0.97) and therefore hold promise for further development as a metabolomics signature of active TB.

These results also demonstrate that when using rapidly developing HRM technology to study the metabolome of persons with infectious diseases, focused analysis of low-abundance metabolites putatively derived from the infecting microorganisms may reveal new diagnostic targets. In our previous analysis, statistical significance was determined by applying a stringent FDR threshold of 5% to all detected metabolites and the PG (16:0_18:1) and Lyso-PI (18:0) molecules described here did not meet that false discovery threshold [7]. However, selecting molecular features by a low *p* alone assumes all features have an equal pre-test probability of being biologically meaningful in the context of the disease process studied [24]. Molecular associations in a select cohort of patients may have little to do with disease pathogenesis and these associations are often difficult to replicate in larger studies [25]. Therefore, integration of prior information relevant to the disease process of interest provides a strategy to help identify more relevant molecular features [25].

For the present study, we hypothesized that previously published metabolites described as specific products of *Mtb* [16] could be detected in plasma and would distinguish active TB patients from household contacts. This approach led to the discovery of two additional metabolites [PG (16:0_18:1) and Lyso-PI (18:0)] with high predictive value for active TB disease that we excluded in our previous study. These metabolites were previously overlooked when correcting for comparisons of all observed chemical features regardless of potential biologic relevance.

Databases commonly used to annotate metabolites in human studies, such as the Kyoto Encyclopedia of Genes and Genomes (KEGG), the Human Metabolome Database (HMDB) and METLIN, contain few metabolites derived from microorganisms [13–15]. Thus, pathogen-derived molecules may be missed or classified as unknowns. To illustrate, in our previous untargeted metabolomics analysis [7], we found that the *m/z* feature 1321.9177 was significantly higher in patients with active TB disease relative to household controls. However, there are no features matching this *m/z* in the METLIN or LIPD MAPS databases, and the metabolite was initially classified as an unmatched (unknown) metabolite. After using an *Mtb*-specific metabolite library, we found this feature matched the mass and retention time of an acylated phosphatidylinositol mannoside found in the cell wall of *Mtb* [16]. Although the chemical identity of this feature could not be verified in our current analysis using tandem MS/MS, its association with TB disease raises an interesting possibility that it may be derived from *Mtb* cell wall.

The current study is the first, to our knowledge, to use HRM to comprehensively screen plasma collected from patients with active TB disease for previously identified *Mtb*-associated metabolites. The ultra-high-resolution of the metabolomics methods used has increased sensitivity to detect very low abundance metabolites in biologic samples [26]. The *Mtb* cell wall is constituted by a wide array of glycolipids, making these ideal targets for biomarkers of active TB disease [16, 17, 27, 28]. It is therefore possible that as HRM technology develops, additional metabolites potentially derived from *Mtb* in persons with active TB disease will be identified.

The metabolites differentiating active TB cases from household contacts in the present study may also reveal insights into the host response to *Mtb* infection. PG (16:0_18:1), for example, has been shown to be a strong activator of diverse natural killer T (dNKT) cells during infection with *Mtb* [29]. While PG (16:0_18:1) is found in the cell wall of *Mtb*, it is also found in human pulmonary surfactant. Presentation of both the bacterial regioisomer [PG (18:1/16:0)], as well as the human regioisomer [PG (16:0/18:1)] by CD1d leads to robust pro-inflammatory responses by dNKT cells [29]. Patients with active TB disease may therefore have concomitantly increased plasma concentrations of both endogenously-derived and *Mtb*-derived PG (16:0_18:1) to augment the host immune response. The relative contribution of human and microbial PG (16:0_18:1) to the overall plasma concentration remains unclear and requires further study.

Similarly, Lyso-PI (18:0) is found in both human cells and in *Mtb*; thus, elevated concentrations of this lipid metabolite may derive from the infecting microorganisms and/or the host response to TB disease. Although the abundance of Lyso-PI (18:0) in human cells is low relative to other glycolipids, it has been described as an important cell signaling molecule, acting through G protein-coupled receptor 55 (GPR55) [30]. Lyso-PI (18:0)-induced activation of GPR55 has been shown to activate nuclear factor of activated T-cells (NFAT) and nuclear factor κ of activated B cells (NF-κB), and has also been associated with activation of wound healing processes in human lung microvascular endothelial cells [31]. Ac1PIM1 is not present in human cells and therefore may be derived from breakdown of the *Mtb* cell wall in the current study. However, this lipid is also found in the cell wall of other types of mycobacteria [27]. Therefore, comparative evaluation of this metabolite in the plasma of individuals during the clinical course of active, symptomatic infection with other mycobacterial species would be of interest.

Although only one of the 16 household contacts in this study had detectable concentrations of two of the three most significant *Mtb* metabolite matches in plasma, half of these individuals had detectable levels of at least one of these metabolites. There are several possible explanations for this finding. Given that household contacts would have been heavily exposed to the active pulmonary TB cases enrolled in the study, some may have had LTBI, which was not tested for in this cohort. There is increasing evidence that persons with LTBI have ongoing organism replication requiring continuing control by the immune system [32, 33], potentially resulting in overlapping plasma metabolic signatures relative to those with active TB. Further investigations are needed to assess the metabolomic profile of individuals with LTBI, targeting the *Mtb*-associated metabolites outlined in this report. Given that PG (16:0_18:1) and lyso-PI (18:0) are also found in human cells and Ac1PIM1 (56:1) is found in the cell wall of other species of mycobacteria [34], future studies should ideally incorporate control groups with non-tuberculous mycobacterial disease and persons with rigorous negative testing for LTBI to further refine the specificity of these metabolites to active TB disease.

This study is subject to several limitations. The sample size for this pilot study was a convenience sample, containing only 17 pulmonary TB cases and 16 household contacts. Due to the small sample size, the three *Mtb*-associated metabolites found to distinguish patients with active TB from their contacts will need to be validated on a larger, prospective cohort of active TB patients and controls with and without LTBI. Plasma HRM studies in humans with active non-tuberculous mycobacterial disease would also be of interest. The current study was conducted at a single center in the country of Georgia with a homogenous (Caucasian) population with a low rate of HIV co-infection. Further work is needed to investigate the presence of these metabolites in other patient populations, including those with HIV co-infection. Additionally, certain *Mtb* lipid metabolites have a larger mass than we queried for identification (i.e. > 2000 *m/z*) and may have been missed by this analysis; also, our plasma extraction method for LC-MS was not optimized for the most hydrophobic lipid species. Finally, while the identities of PG (16:0_18:1) and Lyso-PI (18:0) were supported by MS/MS confirmation experiments, we were unable to generate interpretable fragmentation spectra for Ac1PIM1 (56:1) and this metabolite is therefore reported as a prediction with level five confidence (exact mass of interest) [35]. It is possible the abundance of this metabolite was lower than the threshold necessary to reliably produce interpretable fragmentation spectra.

In future studies, alternative metabolite extraction methods optimized for less polar lipid molecules may increase the yield of *Mtb* lipids in biosamples and allow for additional confirmation of chemical identities. Reproduction of these results in an independent population of control patients both with and without LTBI would also provide additional confidence they are either derived from *Mtb* or related to its pathophysiology. It would also be of interest to analyze patient urine for excretion of these *Mtb* metabolites to better understand the kinetics of detection in relation to TB disease diagnosis and response to therapy. Recent developments using hydrogel nanocage technology suggest the mycobacterial cell wall component lipoarabinomannan can be detected at extremely low concentrations in urine samples [36]. Confirmation of LAM breakdown products in urine, concomitant with LAM, would lend important insight to the utility of this assay as a TB disease biomarker.

In conclusion, we found that among several *Mtb* lipid metabolite *m/z* matches detected, PG (16:0_18:1) and Lyso-PI (18:0) were the molecules most significantly elevated in the plasma of patients with active TB compared to their asymptomatic household contacts. These metabolites provided excellent classification accuracy for active TB. Our pilot data suggests that plasma HRM may hold significant promise for characterization of *Mtb*-associated metabolites in patients with active TB disease. If confirmed in larger studies that include diverse patient populations and relevant controls, further development of the most discriminatory metabolites may inform development of diagnostic biomarkers for active TB.

## Supporting information

S1 File Full feature table. This file contains the full feature table with metabolite intensities for all samples analyzed in this study organized by mass-to-charge ratio (*m/z*), retention time, and minimum and maximum *m/z*.
(ZIP)

S1 Fig MS/MS fragmentation spectra show positive identification of phosphatidylglycerol (16:0_18:1). MS/MS spectra for m/z 747.7. Presence of fragments 673.3, 281.0 and 255.0 in the plasma sample of an active TB patient indicate a match to the [M-H] adduct for phosphatidylglycerol (PG) (16:0_18:1).
(TIFF)

S2 Fig MS/MS fragmentation spectra show positive identification of lysophosphatidylinositol (18:0). MS/MS spectra for m/z 599.5. Presence of fragments 315.0, 283.3 and 152.8 in the plasma sample of an active TB patient indicate a match to the [M-H] adduct for lysophosphatidylinositol (Lyso-PI) (18:0).
(TIFF)
